# Double Consecutive Electrical Countershock for Atrial and Ventricular Fibrillation: A Narrative Critical Review

**DOI:** 10.1111/anec.70251

**Published:** 2026-11-02

**Authors:** Ian Ray, Varun Gopal, Brian Ellis, Abraham Kocheril

**Affiliations:** ^1^ Carle Illinois College of Medicine Urbana Illinois USA; ^2^ Department of Cardiology Sarah Bush Lincoln Health Center Mattoon Illinois USA

**Keywords:** atrial fibrillation, double cardioversion, double defibrillation, double sequential external defibrillation, electrical countershock, ventricular fibrillation

## Abstract

**Background:**

Recent strategies for refractory ventricular fibrillation (VF) and atrial fibrillation (AF) employ two consecutive, biphasic shocks with varying evidence of success. Due to their electrophysiological similarities, the strategies used for VF and AF can be collectively referred to as double consecutive electrical countershock (DCEC). This review aims to identify and synthesize outcome data on the effectiveness of DCEC across both VF and AF.

**Methods:**

This narrative critical review with systematic search methods was conducted in accordance with the PRISMA checklist where applicable. From 672 identified articles, 262 duplicates were removed and 410 screened; 316 articles irrelevant to the study were excluded. Of 94 sought for retrieval, 8 were unavailable and 67 failed full‐text screening, leaving 19 included articles (16 VF, 3 AF). All 19 underwent risk‐of‐bias assessment (RoB2, ROBINS‐I, or AMSTAR‐2) and data extraction.

**Results:**

Full‐scale randomized controlled trials (RCTs) showed low risk of bias, pilot RCTs had “some concerns,” observational studies carried serious risk of bias, and meta‐analyses showed “low confidence.” High‐quality randomized evidence showed improved primary outcomes for DCEC in both refractory VF (survival to discharge, return of spontaneous circulation, good neurological outcome) and high‐BMI AF (arrhythmia termination). Non‐randomized observational studies predominantly showed non‐statistically significant differences.

**Conclusions:**

DCEC shows potential to improve arrhythmia termination in refractory VF and high‐BMI AF. Randomized evidence supports its efficacy, while observational findings remain neutral due to confounding. Success in high‐BMI AF supports elevated BMI as a hypothetical indication for DCEC in VF.

## Graphical abstract

This critical review synthesizes and evaluates evidence on Double Consecutive Electrical Countershock (DCEC) for refractory VF and AF. Utilizing systematic searching and quality appraisal via RoB 2, ROBINS‐I, and AMSTAR‐2, we found high‐quality RCT‐supported efficacy for DSED in refractory VF and dual‐DCCV in high‐BMI AF.

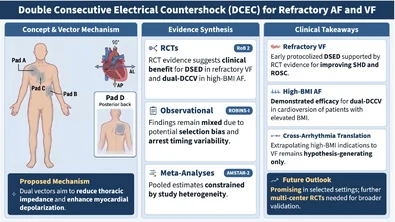

## 1 Introduction

Cardiac arrhythmias such as ventricular fibrillation (VF) and atrial fibrillation (AF) continue to pose major clinical challenges, with VF being a leading cause of sudden cardiac death and AF being the most common sustained arrhythmia (Joglar et al. 2024; Desai and Hajouli 2025). Despite their differences in electrophysiological origin and clinical presentation, both AF and VF are characterized by chaotic myocardial activity that can often be terminated by electrical countershock techniques using a single, biphasic electrical shock (Desai and Hajouli 2025). This technique is called defibrillation for VF and direct current cardioversion for AF. However, in cases where standard single‐shock approaches are unsuccessful, recent strategies have employed a rapid sequence of two biphasic shocks delivered in close succession (Cheskes et al. 2022).

The underlying principle for treating VF and AF with two biphasic shocks is the same: the delivery of two rapid, consecutive shocks from separate electrical sources to overcome high thoracic impedance and achieve more effective myocardial depolarization (Cheskes et al. 2025). For VF, this strategy is called double sequential external defibrillation (DSED), while for AF it is called dual direct‐current cardioversion (dual‐DCCV) (Cheskes et al. 2022; Aymond et al. 2024). VF and AF are usually treated under drastically different circumstances: DSED in the emergency department or in the field, and dual‐DCCV under controlled circumstances in the electrophysiology lab. Therefore, an artificial separation between these similar and comparable interventions has occurred, causing the existing literature to evolve in parallel. To date, studies have only evaluated DSED and dual‐DCCV independently—despite their shared general principles and similar mechanism of action—and no systematic effort has been made to reconcile these approaches.

In this review, we address this literature gap by unifying two‐shock VF and AF interventions under a single term: double consecutive electrical countershock (DCEC). DCEC encompasses both DSED and dual‐DCCV and reflects their shared electrophysiological foundation. As shown in Figure 1, DCEC refers to the delivery of two biphasic, consecutive shocks in close temporal proximity as a therapeutic intervention for unstable arrhythmias.

**FIGURE 1:**
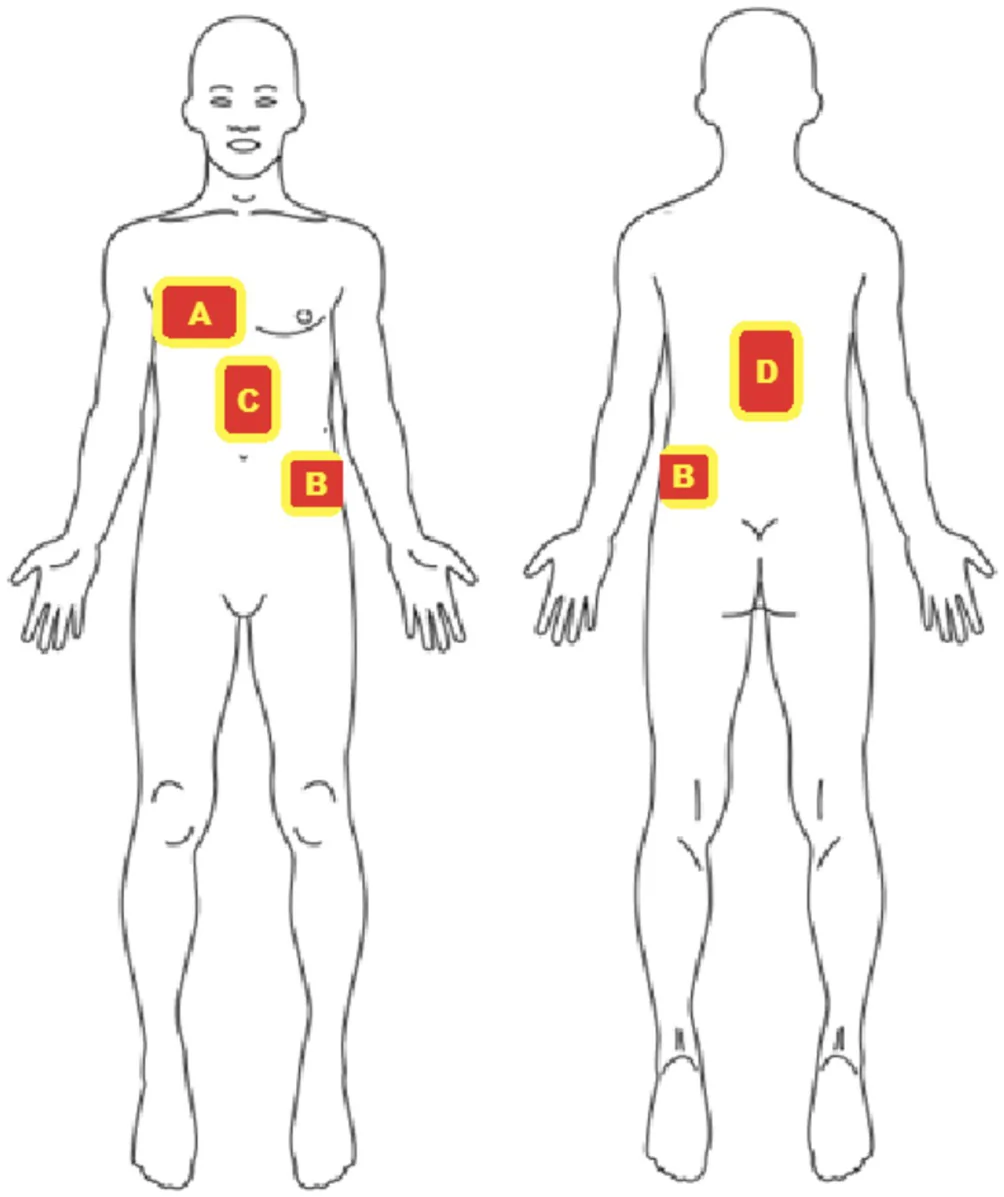
A common placement of four pads for DCEC pairs anterior‐lateral pads (pads A and B) with anterior posterior pads (pads C and D). DCEC proceeds with rapidly sequential shocks on near‐orthogonal vectors, providing depolarization of myocardial tissue from multiple and complementary directions. The above configuration is consistent with current practice guidelines for both DSED and dual‐DCCV (Joglar et al. 2024; Wigginton et al. 2025).

This review aims to identify and synthesize outcome data on the effectiveness of DCEC in human subjects across both VF and AF. It also seeks to bridge an important gap in the literature and explore the potential cross‐applicability of DCEC between these two arrhythmic contexts that have previously been studied in isolation. We aim to evaluate: first, is DCEC more effective than the current standard of care when used in refractory cases? And second, are there specific indications for DCEC that show a greater benefit, such as high body mass index (BMI)?

## 2 Materials and Methods

We conducted a narrative critical review with systematic search methods according to the Preferred Reporting Items for Systematic Reviews and Meta‐Analyses (PRISMA Checklist, Supplementary File S1) checklist where applicable. We conducted a comprehensive search in four databases—PubMed (Appendix A.1), Scopus (Appendix A.2), Embase (Appendix A.3), and CENTRAL (Appendix A.4)—to identify articles relevant to the study. The final search date for inclusion in this study was August 1, 2026. Search records are recorded in Appendix A.

Studies that evaluated the effectiveness of two, rapidly consecutive, therapeutic electrical countershocks on the heart, either in defibrillation or cardioversion procedures, were included in this review. Interventional, observational, and meta‐analysis studies were considered for inclusion. Case studies, non–meta‐analytic systematic reviews, studies involving implanted devices, and studies unavailable in English were excluded.

Database searches identified 672 articles including 262 duplicates, which were removed. The 410 unique articles were screened by title and abstract, and 316 articles irrelevant to the study were eliminated. The remaining 94 articles were sought for retrieval and assessed for eligibility. Eight articles could not be retrieved, and 67 articles did not meet inclusion criteria (Supplementary Table S1). The final 19 articles were included in the review. The review was conducted using the Rayyan, Intelligent Systematic Review software (Ouzzani et al. 2016). Screening was conducted by three independent authors, with two reviewers fully reviewing each article at each step. The study retrieval and selection process is represented in the PRISMA flow diagram in Figure 2.

**FIGURE 2:**
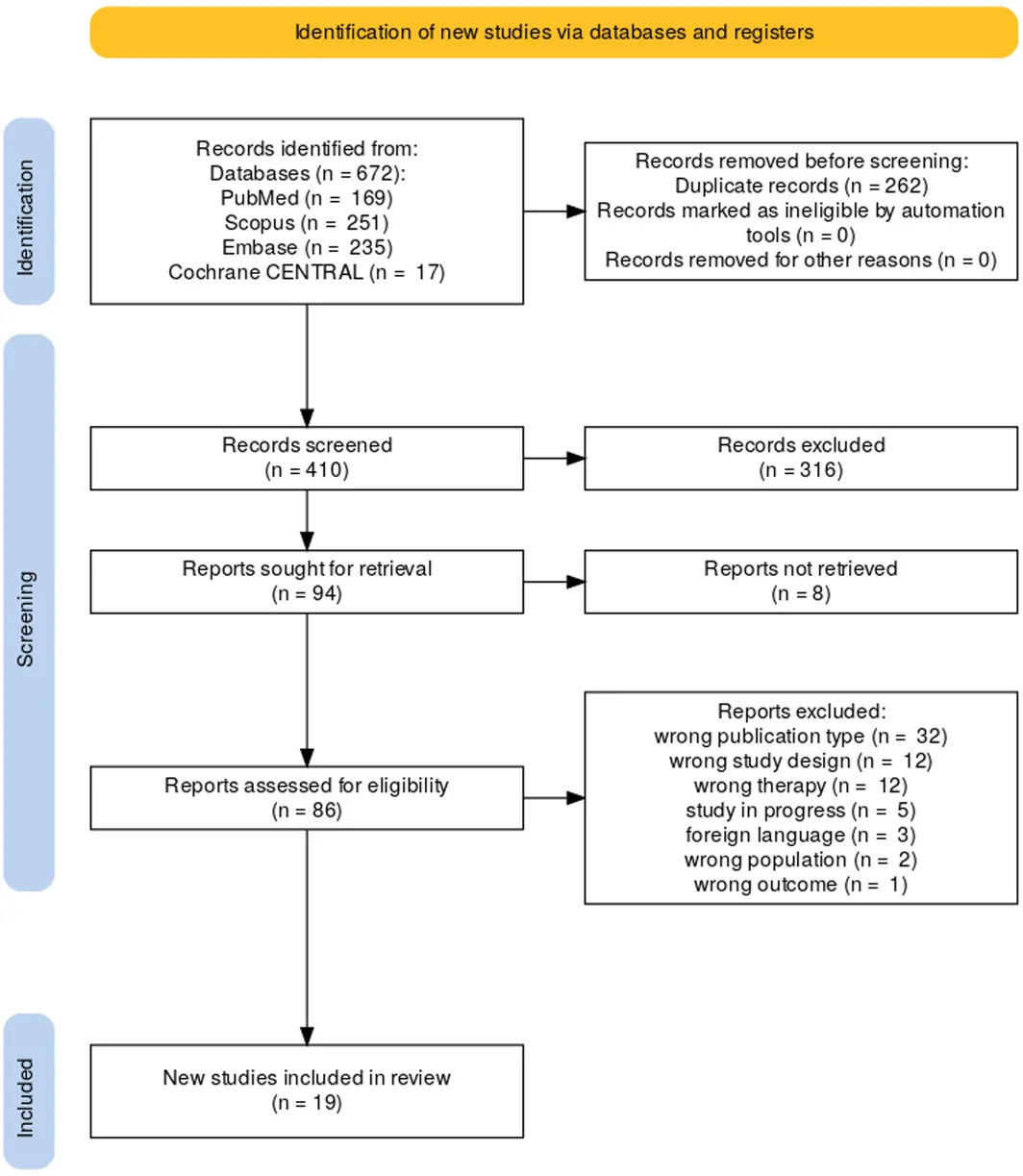
PRISMA diagram, detailing the number of articles included and excluded at each step in the review process.

The 19 articles included in the review were subjected to a risk‐of‐bias assessment, which was conducted independently by two authors according to the RoB2 quality appraisal guidelines for Randomized Controlled Trials (RCT; Appendix B, Table B1), the ROBINS‐I guidelines for observational studies (Appendix B, Table B2), and the AMSTAR‐2 guidelines for meta‐analyses (Appendix B, Table B3) (Sterne et al. 2019; Sterne et al. 2016; Shea et al. 2017). Because this is a narrative critical review informed by systematic search methods, we applied these tools in a streamlined manner rather than using full signaling‐question algorithms. This approach provides appraisal appropriate for the heterogeneous evidence included in this study while avoiding unnecessary complexity beyond the scope of the study.

Data were then extracted from each of the included studies, with primary and secondary outcomes relevant to the arrhythmia studied reported in Table 2. For VF, the primary outcomes were survival to hospital discharge (SHD), return of spontaneous circulation (ROSC), and good neurological outcome (GNO; defined as a modified Rankin scale (mRS) ≤ 2) (Cheskes et al. 2022). Secondary outcomes were survival to hospital admission (SHA), arrhythmia termination (AT), and the occurrence of any adverse events (if recorded). For AF, the primary outcome was AT. Secondary outcomes were first‐shock success and the occurrence of any complications.

## 3 Results

Data were extracted from 19 articles (16 VF, 3 AF). Study types included: retrospective cohort study (8), systematic review and meta‐analysis (4), RCT (2), RCT pilot study (2), RCT secondary analysis (1), prospective crossover (1), and case‐control (1). Overall risk of bias varied significantly by study design (Table 1). Both full‐scale RCTs had low overall risk of bias (RoB2), while pilot RCTs showed “some concerns.” All observational (cohort, case‐control, etc.) studies carried significant risk of bias (ROBINS‐I), generally due to significant confounding and selection bias. Many observational studies also lacked randomization. Finally, for all four included meta‐analyses a determination of “low confidence” in the results was made (AMSTAR‐2). Individual study characteristics and outcome metrics are displayed in Table 2.

**TABLE 1:** A unified critical appraisal of the studies included in the review. Studies are ordered chronologically within the subcategory of tool used.

Study	Arrhythmia	Design	Tool used	Key domains with concerns	Overall risk‐of‐bias judgment
Riva et al. (2026)	VF	RCT Pilot	RoB2	Unblinded, small sample limitations	Some concerns
Aymond et al. (2024)	AF	RCT	RoB2	Unblinded, minimal protocol deviations	Low
Cheskes et al. (2022)	VF	RCT	RoB2	Unblinded	Low
Cheskes et al. (2020)	VF	RCT Pilot	RoB2	Unblinded, small sample limitations	Some concerns
Dicker et al. (2026)	VF	Retrospective Cohort	ROBINS‐I	Major confounding, considerable selection bias	Serious
Cheskes et al. (2025)	VF	Retrospective Cohort	ROBINS‐I	Major confounding, considerable selection bias, no randomization	Serious
Cheskes et al. (2024)	VF	RCT Secondary Analysis	ROBINS‐I	Major confounding, considerable selection bias, no randomization	Serious
Kim et al. (2020)	VF	Retrospective Cohort	ROBINS‐I	Major confounding, considerable selection bias, no randomization	Serious
Beck et al. (2019)	VF	Retrospective Cohort	ROBINS‐I	Major confounding, considerable selection bias, no randomization	Serious
Gardner et al. (2019)	AF	Case–Control	ROBINS‐I	Major confounding, considerable selection bias, no randomization	Serious
Cheskes et al. (2019)	VF	Retrospective Cohort	ROBINS‐I	Major confounding, considerable selection bias, no randomization	Serious
Mapp et al. (2019)	VF	Retrospective Cohort	ROBINS‐I	Major confounding, some selection bias, no randomization	Serious
Emmerson et al. (2017)	VF	Retrospective Cohort	ROBINS‐I	Major confounding, considerable selection bias, no randomization	Serious
Ross et al. (2016)	VF	Retrospective Cohort	ROBINS‐I	Major confounding, considerable selection bias, no randomization	Serious
Bjerregaard et al. (1999)	AF	Prospective Crossover	ROBINS‐I	Major confounding, considerable selection bias, no randomization	Serious
Yu et al. (2024)	VF	Systematic Review/Meta‐Analysis	AMSTAR‐2	No registered protocol or assessment for publication bias, RoB not considered in interpretation	Low Confidence
Abuelazm et al. (2023)	VF	Systematic Review/Meta‐Analysis	AMSTAR‐2	No registered protocol or assessment for publication bias, RoB not considered in interpretation	Low Confidence
Miraglia et al. (2020)	VF	Systematic Review/Meta‐Analysis	AMSTAR‐2	No registered protocol or assessment for publication bias, RoB not considered in interpretation	Low Confidence
Delorenzo et al. (2019)	VF	Systematic Review/Meta‐Analysis	AMSTAR‐2	No registered protocol or assessment for publication bias, RoB not considered in interpretation	Low Confidence

**TABLE 2:** Summary data from articles included in this study.

Study	Design	Country	*N*	Arr.	Outcomes (RR/OR; 95% CI)
Riva et al. (2026)	Pilot RCT	Sweden	40	VF	– SHD: NSSD (RR 4.55; 0.67–30.7) – ROSC: NSSD (RR 1.33; 0.7–2.4) – SHA: NSSD (RR 1.71; 0.8–3.8)
Aymond et al. (2024)	RCT	USA	200	AF	– AT: Greater for dual‐DCCV (OR 6.7; 3.3–13.6) – First shock success: Greater for dual‐DCCV (OR 8.0; 1.8–36.4) – No complications observed
Cheskes et al. (2022)	RCT	Canada	405	VF	– SHD: Greater for DSED (RR 2.21; 1.3–3.7) – ROSC: Greater for DSED (RR 1.72; 1.2–2.4) – GNO: Greater for DSED (RR 2.21; 1.3–3.9) – SHA: Greater for DSED (RR 1.72; 1.2–2.4) – AT: Greater for DSED (RR 1.25; 1.1–1.4)
Cheskes et al. (2020)	Pilot RCT	Canada	152	VF	– SHD: NSSD (RR 1.21; 0.7–2.2) – ROSC: NSSD (RR 1.55; 0.9–2.8) – GNO: NSSD (RR 1.16; 0.6–2.4) – SHA: NSSD (RR 1.31; 0.9–2.0) – AT: NSSD (RR 1.16; 0.9–1.5)
Dicker et al. (2026)	Retrospective Cohort	New Zealand	1401	VF	– SHD: NSSD for early DSED (up to 3rd shock) (OR 1.05; 0.6–2.0) – ROSC: NSSD for early DSED (OR 1.05; 0.6–1.8) – SHA: lower in DSED (OR 0.55; 0.4–0.8)
Cheskes et al. (2025)	Retrospective Cohort	Canada	342	VF	– SHD: Greater for DSED (OR 3.12; 1.6–6.3) – ROSC: Greater for DSED (OR 3.81; 2.2–6.6) – AT: Failure to terminate greater with conventional defibrillation (OR 0.62; 0.4–0.9)
Cheskes et al. (2024)	RCT Secondary Analysis	Canada	345	VF	– SHD: Greater for DSED in overall cohort (OR 2.18; 1.1–4.5) – ROSC: Greater for DSED in overall cohort (OR 2.19; 1.3–3.8), NSSD for refractory VF cohort (OR 4.40; 0.7–28.8) – GNO: Greater for DSED in overall cohort (OR 2.56; 1.2–5.5) – AT: Greater for DSED in overall cohort (OR 2.10; 1.1–4.0), NSSD for refractory VF cohort (OR 0.74; 0.2–3.1)
Kim et al. (2020)	Retrospective Cohort	Rep. of Korea	38	VF	– SHD: NSSD (RR 2.88; 0.9–9.4) – SHA: higher in DSED (RR 2.88; 1.4–5.9) – GNO: NSSD (RR 3.09; 0.7–13.9)
Beck et al. (2019)	Retrospective Cohort	USA	310	VF	– SHD: NSSD (OR 0.46; 0.3–1.5) – ROSC: lower in DSED (OR 0.46; 0.3–0.9) – SHA: NSSD (OR 0.57; 0.3–1.1)
Gardner et al. (2019)	Case–Control	USA	77	AF	– AT: NSSD (OR 0.57; 0.3–1.2) – BMI was found to be a risk factor for failure of standard cardioversion and the need for dual‐DCCV – One episode of transient hypoxia observed
Cheskes et al. (2019)	Retrospective Cohort	Canada	252	VF	– ROSC: NSSD overall (RR 0.8; 0.4–1.6), but greater for DSED on defib attempts 4–8 (RR 1.8; 1.4–5.9) – AT: NSSD overall (RR 1.0; 0.8–1.2), but greater in DSED on defib attempts 4–8 (RR 1.7; 1.1–2.6)
Mapp et al. (2019)	Retrospective Cohort	USA	205	VF	– SHA: NSSD (RR 0.91; 0.4–2.1)
Emmerson et al. (2017)	Retrospective Cohort	UK	45	VF	– SHD: NSSD (RR 1.00; 0.2–5.2) – ROSC: NSSD (RR 1.00; 0.4–2.4)
Ross et al. (2016)	Retrospective Cohort	USA	279	VF	– SHD: NSSD (RR 0.52; 0.2–1.5) – ROSC: NSSD (RR 0.65; 0.3–1.3) – GNO: NSSD (RR 0.50; 0.2–1.7) – SHA: NSSD (RR 0.74; 0.5–1.7)
Bjerregaard et al. (1999)	Prospective Crossover	USA	24	AF	– AT: NSSD (OR 3.33; 0.9–11.8) – No complications observed
Yu et al. (2024)	Systematic Review/Meta‐Analysis	China	1360	VF	– SHD: NSSD (RR 0.91; 0.5–1.8) – ROSC: lower in DSED compared to SD if simultaneous (RR 0.65; 0.5–0.9), NSSD if sequential (RR 1.00; 0.7–1.4)
Abuelazm et al. (2023)	Systematic Review/Meta‐Analysis	Egypt	1632	VF	– SHD: NSSD (OR 1.14; 0.6–2.4) – ROSC: NSSD (OR 0.81; 0.4–1.5) – GNO: NSSD (OR 1.35; 0.5–4.0) – SHA: NSSD (OR 1.12; 0.6–2.2)
Miraglia et al. (2020)	Systematic Review/Meta‐Analysis	USA	1061	VF	– SHD: NSSD (OR 0.66; 0.4–1.2) – ROSC: NSSD (OR 0.68; 0.4–1.0) – SHA: NSSD (OR 0.77; 0.5–1.2)
Delorenzo et al. (2019)	Systematic Review/Meta‐Analysis	Australia	5351	VF	– SHD: NSSD (RR 0.69; 0.3–1.6) – ROSC: NSSD (RR 0.86; 0.5–1.5) – SHA: NSSD (RR 0.98; 0.6–1.6)

Abbreviations: AT, arrhythmia termination; GNO, good neurological outcome; NSSD, not statistically significant difference; ROSC, return of spontaneous circulation; SHA, survival to hospital admission; SHD, survival to hospital discharge.

### 3.1 Ventricular Fibrillation

For VF, evaluated primary outcomes included survival to hospital discharge (SHD), return of spontaneous circulation (ROSC), and good neurological outcome (mRS ≤ 2; GNO); while secondary outcomes included survival to hospital admission (SHA) and arrhythmia termination (AT). The high‐level randomized evidence supported DSED as a superior therapy compared to standard defibrillation. In particular, the DOSE‐VF trial (Cheskes et al. 2022) observed more than double the likelihood of SHD (RR 2.21; 95% CI 1.3–3.7) and GNO (RR 2.21; 95% CI 1.3–3.9), alongside significantly higher rates of ROSC (RR 1.72; 95% CI 1.2–2.4) and AT (RR 1.25; 95% CI 1.1–1.4). Secondary analysis of this same cohort (Cheskes et al. 2024) further bolstered these results with DSED showing significant benefit for SHD, ROSC, GNO, and AT.

In contrast, non‐randomized observational studies and meta‐analyses predominantly reported non‐statistically significant differences between DSED and standard defibrillation for SHD, ROSC, and SHA. One protocol‐specific cohort study (Cheskes et al. 2019) also found timing‐dependent efficacy, showing improved ROSC and AT when DSED was initiated during shock attempts 4–8. Another retrospective cohort reported significantly lower ROSC associated with unstandardized prehospital DSED (Beck et al. 2019), and one systematic review (Yu et al. 2024) noted reduced ROSC specifically when shocks were delivered simultaneously rather than sequentially.

### 3.2 Atrial Fibrillation

For AF, the evaluated primary outcome was AT, while secondary outcomes were first‐shock success and procedural complications.

The only randomized evaluation (Aymond et al. 2024) supported dual‐DCCV as a superior therapy compared to standard cardioversion in terms of AT (OR 6.7; 95% CI 3.3–13.6) and first‐shock success (OR 8.0; 95% CI 1.8–36.4). Zero adverse events occurred. Notably, this trial was restricted to patients with elevated BMI (≥ 35). Conversely, non‐randomized observational studies in general or refractory AF populations (Gardner et al. 2019; Bjerregaard et al. 1999) showed non‐statistically significant differences in AT for dual‐DCCV compared to standard cardioversion.

## 4 Discussion

Double consecutive electrical countershock (DCEC) represents a unified electrophysiological approach for terminating either VF or AF. Although this technique is referred to as double sequential external defibrillation (DSED) for VF and dual direct‐current cardioversion (dual‐DCCV) for AF, both interventions require the application of rapid, consecutive shocks across alternating vectors to overcome thoracic impedance and depolarize the myocardium (Cheskes et al. 2022; Aymond et al. 2024). Therefore, these therapies may be evaluated under the shared framework of DCEC, allowing for consistent insights regarding efficacy and patient‐specific indications to emerge.

### 4.1 Study Design Differences Explain Variation in VF Outcomes

The clear discrepancy in the VF literature between supportive randomized trials and neutral or sometimes critical observational studies can be explained by the differences in study design and subsequent confounding. High‐quality randomized evidence, particularly the DOSE‐VF trial, demonstrated significant clinical benefits for DSED in refractory VF as evidenced by significantly improved survival to hospital discharge (SHD), return of spontaneous circulation (ROSC), good neurological outcome (GNO), survival to hospital admission (SHA), and arrhythmia termination (AT) (Cheskes et al. 2022). This study utilized standardized protocols to ensure early, consistent delivery of DSED after only three failed shock attempts, minimizing time to intervention and providing clearer evidence of the efficacy of DSED.

Conversely, observational studies predominantly reported non‐statistically significant differences or, in isolated reports (Beck et al. 2019), worsened outcomes for DSED when compared to standard defibrillation. This pattern can be largely attributed to the presence of significant confounding and selection bias in most of the reports, as highlighted in our ROBINS‐I risk‐of‐bias assessment. In the context of observational studies, DSED was typically applied by paramedics as an unstandardized salvage therapy in prolonged, unwitnessed, and deeply refractory cardiac arrests. Consequently, the true therapeutic effect of DSED is systematically masked in these observational studies by the timing and context of the intervention. In fact, in the one observational cohort that specifically analyzed timing (Cheskes et al. 2019), escalation to DSED at a comparatively early point in cardiac arrest management (shocks 4–8) demonstrated improved ROSC and AT compared to standard defibrillation.

Similarly to the observational studies, the pooled effect estimates reported across the included meta‐analyses were heavily influenced by variation in DSED protocols. Our AMSTAR‐2 risk‐of‐bias assessment demonstrated low overall confidence across the included meta‐analyses, primarily due to lack of risk‐of‐bias assessments and strategies to account for publication bias. Therefore, pooled cohorts were simply integrated into the meta‐analyses alongside RCT data without risk‐of‐bias weighting, causing the same errors present in the observational studies to permeate the meta‐analyses. Furthermore, some meta‐analyses predated the publication of the DOSE‐VF trial, while the more recent meta‐analyses (Yu et al. 2024; Abuelazm et al. 2023) heavily diluted the data from the randomized trials with observational data when performing their analyses. Additionally, though Yu et al. found simultaneous shocks to be less effective than standard defibrillation while sequential shocks were found to have no statistically significant difference from standard defibrillation, the actual methodology was almost always consecutive shocks in both cases because the technique involved human operation without automation equipment (Hamilton et al. 2020). Therefore, the subgroup differences should be reviewed and interpreted with caution.

Finally, a notable limitation of the current evidence base for VF is that the definitive randomized trial data and secondary analyses originate from a single research collaborative in Toronto, Canada (Cheskes et al.). Although this group utilized rigorous methodology and performed well on the risk‐of‐bias assessments in this study, there is clear need for independent, multi‐center replication of this study to establish external validity across diverse EMS systems. Emerging pilot randomized trials, such as the double‐D trial in Sweden (Riva et al. 2026), suggest that international replication is feasible and provide the groundwork for further studies.

### 4.2 Elevated BMI May Be a Potential Indication for DCEC

In contrast to the emergency field setting of DSED, dual‐DCCV for AF is typically conducted in the more controlled environment of an electrophysiology lab. Randomized evidence on AF demonstrates significant efficacy for dual‐DCCV in comparison to standard cardioversion. A trial specifically targeting patients with obesity (BMI ≥ 35; Aymond et al. 2024) found dual‐DCCV to have nearly a seven‐fold increase in AT in comparison to standard cardioversion (OR 6.7; 95% CI 3.3–13.6). There was also a significant increase in the first‐shock success for dual‐DCCV relative to standard, single‐shock cardioversion. These findings were further supported by those of Gardner et al., which found that increased BMI is a risk factor for the necessity of DCEC to achieve AF termination (Table 2) (Gardner et al. 2019).

A clear physiological basis underlies these findings: elevated BMI increases thoracic impedance, leading to a reduction in current delivery to the myocardium and greater energy requirements for successful arrhythmia termination (Heyer et al. 2022). This greater energy requirement is likely the fundamental reason why multiple shocks are more effective in high BMI patients and explains the greater failure rates of single‐shock cardioversion relative to dual‐DCCV (Aymond et al. 2024; Gardner et al. 2019; Heyer et al. 2022). By delivering two rapid shock sequences across dual anatomical vectors, DCEC effectively mitigates thoracic impedance, increases current density, increases the amount of energy applied to the myocardium, and increases the likelihood of termination for both arrhythmias (Aymond et al. 2024; Heyer et al. 2022).

Given the promise of dual‐DCCV/DCEC as a first‐line treatment for AF in high BMI patients, we propose further research be conducted to confirm the findings of Aymond et al. (2024) and Gardner et al. (2019). Furthermore, since the DCEC techniques used for AF and VF termination involve similar electrophysiology, we propose that elevated BMI be considered as a potential indication for DSED/DCEC in the termination of VF in future trials. Current protocols primarily indicate DCEC for the 3rd shock in refractory VF treatment, but given the success of DCEC in AF treatment, there is potential for improved outcomes in high BMI patients with VF, and this should be explored. However, no currently available evidence directly supports elevated BMI as an indication for DSED/DCEC in VF termination and clinical practice should not be modified based on this postulation. DCEC for VF must be studied independently because of the differences in whole‐body physiology at the time of DCEC application, potentially leading to variations in efficacy and increased adverse outcomes.

## 5 Conclusions

This review demonstrates the potential of DCEC to improve arrhythmia termination rates in both AF and VF. While observational data remain mixed due to confounding and selection bias, high‐quality randomized trial evidence demonstrates clinical benefit for DSED in refractory VF and dual‐DCCV in high‐BMI AF.

Potential exists for these two distinct uses of DCEC to inform one another, particularly in the technique's success in terminating AF in high BMI patients at a greater rate than standard cardioversion. Consequently, there is potential for high BMI to be an indication for the use of DCEC in the treatment of VF as well. However, extrapolating high‐BMI indications from AF to VF remains hypothesis‐generating only, and further multi‐center randomized trials are necessary to explore the clinical validity and utility of our hypothesis.

## Author Contributions

**Ian Ray:** conceptualization, data curation, formal analysis, investigation, methodology, project administration, resources, software, supervision, validation, visualization, writing – original draft, writing – review and editing. **Varun Gopal:** conceptualization, data curation, formal analysis, investigation, methodology, resources, software, validation, visualization, writing – original draft, writing – review and editing. **Brian Ellis:** conceptualization, data curation, formal analysis, investigation, methodology, resources, software, supervision, validation, visualization, writing – original draft, writing – review and editing. **Abraham Kocheril:** conceptualization, supervision, validation, writing – review and editing.

## Funding

The authors have nothing to report.

## Conflicts of Interest

The authors declare no conflicts of interest.

## Supporting information


**Supplementary File S1:** PRISMA checklist.

**Supplementary Table S1:** Studies excluded at the full‐text stage with exclusion justifications.

## Data Availability

All data supporting this review are available in the cited publications.

## Appendix A Searching Records

### A.1 PubMed Search Records

PubMed Search 1: Query: ("double" OR "dual" OR "sequential") AND ("defibril*" OR "cardiovers*") AND (ventricular fibrill* OR dsed OR atrial fibrill*) Results: 1,107

PubMed Search 2: Query: ("double" OR "dual" OR "sequential" OR "stepwise" OR "simultaneous" OR "orthogonal" OR "quadruple") AND ("defibril*" OR "cardiovers*") AND (ventricular fibrill* OR dsed OR atrial fibrill*) Results: 1,366

PubMed Search 3: Query: ("double" OR "dual" OR "sequential" OR "stepwise" OR "simultaneous" OR "orthogonal" OR "quadruple") AND ("defibril*" OR "cardiovers*") AND (ventricular fibrill* OR dsed OR atrial fibrill*) NOT "cardioverter*"[ti] Results: 1,122

PubMed Search 4: Query: ("double"[tiab] OR "dual"[tiab] OR "sequential"[tiab] OR "stepwise"[tiab] OR "simultaneous"[tiab] OR "orthogonal"[tiab] OR "quadruple"[tiab]) AND ("defibril*" OR "cardiovers*") AND ("ventricular fibrill*"[tiab] OR "dsed"[tiab] OR "atrial fibrill*"[tiab]) NOT "cardioverter*"[ti] Results: 923

PubMed Search 5: Query: ("double"[tiab] OR "dual"[tiab] OR "sequential"[tiab] OR "stepwise"[tiab] OR "simultaneous"[tiab] OR "orthogonal"[tiab] OR "quadruple"[tiab]) AND ("defibril*" OR "cardiovers*") AND ("ventricular fibrill*"[tiab] OR "dsed"[tiab] OR "atrial fibrill*"[tiab]) NOT ("cardioverter*"[ti] OR "ablation*"[ti]) Results: 869

PubMed Search 6: Query: ("double defibrillation"[tiab: 4] OR "dual defibrillation"[tiab: 4] OR "sequential defibrillation"[tiab: 4] OR "simultaneous defibrillation"[tiab: 4] OR "stepwise cardioversion"[tiab: 4] OR "simultaneous cardioversion"[tiab: 4] OR "orthogonal cardioversion"[tiab: 4] OR "quadruple cardioversion"[tiab: 4] OR "double cardioversion"[tiab: 4] OR "dual cardioversion"[tiab: 4]) AND ("ventricular fibril*"[tiab] OR "dsed"[tiab] OR "atrial fibrill*"[tiab]) NOT ("cardioverter*"[ti] OR "ablation*"[ti]) Results: 143

PubMed Search 7: Query: ("double shocks"[tiab: 4] OR "sequential shocks"[tiab: 4] OR "double defib rillation"[tiab: 4] OR "dual defibrillation"[tiab: 4] OR "sequential defibrillation"[tiab: 4] OR "simultaneous defibrillation"[tiab: 4] OR "stepwise cardioversion"[tiab: 4] OR "simultaneous cardioversion"[tiab: 4] OR "orthogonal cardioversion"[tiab: 4] OR "quadruple cardioversion"[tiab: 4] OR "double cardioversion"[tiab: 4] OR "dual cardioversion"[tiab: 4]) AND ("ventricular fibril*"[tiab] OR "dsed"[tiab] OR "atrial fibrill*"[tiab]) NOT ("cardioverter*"[ti] OR "ablation*"[ti]) Results: 163

PubMed Search 8: Query: ("double shocks"[tiab: 4] OR "sequential shocks"[tiab: 4] OR "double defib rillation"[tiab: 4] OR "dual defibrillation"[tiab: 4] OR "sequential defibrillation"[tiab: 4] OR "simultaneous defibrillation"[tiab: 4] OR "stepwise cardioversion"[tiab: 4] OR "simultaneous cardioversion"[tiab: 4] OR "orthogonal cardioversion"[tiab: 4] OR "quadruple cardioversion"[tiab: 4] OR "double cardioversion"[tiab: 4] OR "dual cardioversion"[tiab: 4]) AND ("ventricular fibril*"[tiab] OR "dsed"[tiab] OR "atrial fibrill*"[tiab]) NOT ("cardioverter*"[ti] OR "ablation*"[ti]) NOT dogs[MeSH] Results: 155

PubMed Search 9: Query: ("double shocks"[tiab: ~3] OR "sequential shocks"[tiab: ~3] OR "double defibrillation"[tiab: ~3] OR "dual defibrillation"[tiab: ~3] OR "sequential defibrillation"[tiab: ~3] OR "simultaneous defibrillation"[tiab: ~3] OR "stepwise cardioversion"[tiab: ~3] OR "simultaneous cardioversion"[tiab: ~3] OR "orthogonal cardioversion"[tiab: ~3] OR "quadruple cardioversion"[tiab: ~3] OR "double cardioversion"[tiab: ~3] OR "dual cardioversion"[tiab: ~3]) AND ("ventricular fibril*"[tiab] OR "dsed"[tiab] OR "atrial fibrill*"[tiab]) NOT ("cardioverter*"[ti] OR "ablation*"[ti]) NOT (swine[MeSH] OR dogs[MeSH])

Initial Results (January 2025): 133

Updated Results (August 2025): 148

Updated Results (December 2025): 154

Updated Results (August 2026): 169

### A.2 Scopus Search Records

Scopus Search 1: Query: TITLE‐ABS‐KEY (("double" OR "dual" OR "sequential" OR "stepwise" OR "simultaneous" OR "orthogonal" OR "quadruple") AND ("defibril*" OR "cardiover*") AND (ventricular AND fibrill* OR dsed OR atrial AND fibrill*)) Results: 1,452

Scopus Search 2: Query: TITLE‐ABS‐KEY (("double" OR "dual" OR "sequential" OR "stepwise" OR "simultaneous" OR "orthogonal" OR "quadruple") W/4 ("defibril*" OR "cardiover*") AND (ventricular fibrill* OR dsed OR atrial fibrill*)) Results: 400

Scopus Search 3: Query: TITLE‐ABS‐KEY (("double" OR "dual" OR "sequential" OR "stepwise" OR "simultaneous" OR "orthogonal" OR "quadruple") W/4 ("defibril*" OR "cardiover*") AND (ventricular AND fibrill* OR dsed OR atrial AND fibrill*)) AND NOT TITLE ("cardioverter" OR "ablation") Results: 307

Scopus Search 4: Query: TITLE‐ABS‐KEY (("double" OR "dual" OR "sequential" OR "stepwise" OR "simultaneous" OR "orthogonal" OR "quadruple") W/3 ("defibril*" OR "cardiover*") AND (ventricular AND fibrill* OR dsed OR atrial AND fibrill*)) AND NOT TITLE ("cardioverter" OR "ablation") Results: 291

Scopus Search 5: Query: TITLE‐ABS‐KEY (("double" OR "dual" OR "sequential" OR "stepwise" OR "simultaneous" OR "orthogonal" OR "quadruple") PRE/3 ("defibril*" OR "cardiover*") AND (ventricular AND fibrill* OR dsed OR atrial AND fibrill*)) AND NOT TITLE ("cardioverter" OR "ablation") Results: 250

Scopus Search 6: Query: TITLE‐ABS‐KEY (("double" OR "dual" OR "sequential" OR "stepwise" OR "simultaneous" OR "orthogonal" OR "quadruple") PRE/3 ("defibril*" OR "cardiover*") AND (ventricular AND fibrill* OR dsed OR atrial AND fibrill*)) AND NOT TITLE ("cardioverter" OR "ablation" OR “dual chamber”) Results: 223

Scopus Search 7: Query: TITLE‐ABS‐KEY (("double" OR "dual" OR "sequential" OR "stepwise" OR "simultaneous" OR "orthogonal" OR "quadruple") PRE/3 ("defibril*" OR "cardiover*") AND (ventricular AND fibrill* OR dsed OR atrial AND fibrill*)) AND NOT TITLE ("cardioverter" OR "ablation" OR “dual chamber”) AND NOT TITLE‐ABS ("dog" OR "swine")

Initial Results (January 2025): 211

Updated Results (August 2025): 226

Updated Results (December 2025): 233

Updated Results (August 2026): 251

### A.3 Embase Search Records

Embase Search 1: Query: ((('double' NEAR/4 'shocks'):ti,ab,kw) OR (('sequential' NEAR/4 'shocks'):ti,ab,kw) OR (('double' NEAR/4 'defibrillation'):ti,ab,kw) OR (('dual' NEAR/4 'defibrillation'):ti,ab,kw) OR (('sequential' NEAR/4 'defibrillation'):ti,ab,kw) OR (('simultaneous' NEAR/4 'defibrillation'):ti,ab,kw) OR (('stepwise' NEAR/4 'cardioversion'):ti,ab,kw) OR (('simultaneous' NEAR/4 'cardioversion'):ti,ab,kw) OR (('or' NEAR/4 'thogonal' NEAR/4 'cardioversion'):ti,ab,kw) OR (('quadruple' NEAR/4 'cardioversion'):ti,ab,kw) OR (('double' NEAR/4 'cardioversion'):ti,ab,kw) OR (('dual' NEAR/4 'cardioversion'):ti,ab,kw)) AND ('ventricular fibril*':ti,ab,kw OR 'dsed':ti,ab,kw OR 'atrial fibrill*':ti,ab,kw) NOT ('cardioverter*':ti OR 'ablation*':ti) NOT ('pig'/exp OR 'dog'/exp)

Initial Results (August 2026): 235

### A.4 CENTRAL Search Records

CENTRAL Search 1: Query: ("double shocks" OR "sequential shocks" OR "double defibrillation" OR "dual defibrillation" OR "sequential defibrillation" OR "simultaneous defibrillation" OR "stepwise cardioversion" OR "simultaneous cardioversion" OR "orthogonal cardioversion" OR "quadruple cardioversion" OR "double cardioversion" OR "dual cardioversion"):ti,ab,kw AND ((ventricular NEXT fibril*) OR "dsed" OR (atrial NEXT fibril*)):ti,ab,kw NOT (cardioverter*:ti OR ablation*:ti) NOT ([mh "swine"] OR [mh "dogs"])

Initial Results (August 2026): 17

## Appendix B Quality Appraisal/Risk‐of‐Bias Assessment Tables

**TABLE B1:** RoB2 quality appraisal of included randomized controlled trials.

Study	Randomization	Deviations from intended interventions	Missing outcome data	Outcome measurement	Selective reporting	Overall RoB judgment
Riva et al. (2026)	Randomization described; baseline characteristics balanced	Unblinded EMS; minor deviations due to field conditions	Outcome data complete for ROSC, survival to admission, survival to 30 days, good neuro outcomes (measured 2 ways), some small sample limitations	Objective outcomes (ROSC, survival to admission, survival to 30 days, good neuro outcomes (measured 2 ways)) defined and measured consistently	Pilot protocol available, all prespecified outcomes reported	Some concerns
Aymond et al. (2024)	Randomization described; baseline characteristics balanced; slight age deviation	Unblinded clinicians; minimal deviations in shock delivery protocol	Outcome data complete with all randomized patients analyzed	Objective outcomes (cardioversion success, arrhythmias, chest pain, MI, stroke) measured consistently	Protocol available, all outcomes reported	Low
Cheskes et al. (2022)	Cluster randomization described; baseline characteristics balanced	Unblinded EMS; minimal deviations from shock protocols	Outcome data complete for survival to hospital discharge, ROSC, VF termination, and good neuro outcomes	Objective outcomes (ROSC, VF termination, survival to discharge, good neuro outcomes) were defined and measured consistently	Protocol registered and available, all prespecified outcomes reported	Low
Cheskes et al. (2020)	Cluster randomization described, but small sample increases risk	Unblinded EMS; interventions delivered per protocol but subject to EMS discretion	Outcome data complete for ROSC and VF termination	Objective outcomes (ROSC, VF termination) were measured consistently	All reported outcomes appear prespecified, protocol detail is limited	Some concerns

**TABLE B2:** ROBINS‐I quality appraisal of included observational studies. None of the included studies had any evidence of selective reporting, so that column has been omitted.

Study	Confounding	Selection bias	Classification of interventions	Deviations from intended interventions	Missing data	Outcome measurement	Overall ROBINS‑I judgment
Dicker et al. (2026)	Major: DSED only used in refractory cases	Retrospective, no random assignment to DSED or standard	Intervention classification clear, protocol well described	EMS unblinded, field conditions lead to variations	Outcome data complete	Objective outcomes (ROSC, 30‐day survival) measured consistently	Serious
Cheskes et al. (2025)	Major: strategy chosen based on level of VF refractoriness and paramedic judgment	Retrospective EMS selection; no random assignment to shock strategy	Intervention classification clear: standard, VC, and DSED	EMS unblinded, field conditions lead to variations	Outcome data complete	Objective outcomes (time in VF, ROSC, survival to hospital discharge) measured consistently	Serious
Cheskes et al. (2024)	Major: escalation to VC or DSED only occurred in refractory or recurrent VF	Re‐grouping breaks randomization, EMS crews used clinical judgment	Intervention classification clear: standard, VC, and DSED	EMS unblinded, field conditions lead to variations	Outcome data complete	Objective outcomes (survival to hospital discharge, VF termination, ROSC, and neuro outcomes) measured consistently	Serious
Kim et al. (2020)	Major: DSED only used in refractory cases	Retrospective EMS selection; no random assignment to shock strategy	Intervention classification clear, protocol well described	EMS unblinded, field conditions lead to variations	Some outcome data missing, nothing catastrophic	Objective outcomes (survival to discharge, survival to admission, good neurological outcome) measured consistently	Serious
Beck et al. (2019)	Major: DSED used selectively in the most refractory VF/VT cases	Retrospective EMS selection; no random assignment to shock strategy	Intervention classification clear, protocol well described	EMS unblinded, field conditions lead to variations	Outcome data largely complete; minimal missing data on ROSC and VF termination	Objective outcomes (ROSC, survival to hospital admission/discharge, survival to 72 h) measured consistently	Serious
Gardner et al. (2019)	Major: Dual External DCCV used selectively for prior failed cardioversion or high BMI	Retrospective selection, decisions based on clinician judgment	Intervention classification clear, protocol well described	Minimal deviations from protocol, performed in well‐controlled EP lab	Outcome data complete	Objective outcome (cardioversion success) measured consistently	Serious
Cheskes et al. (2019)	Major: DSED only used in refractory cases	Retrospective EMS selection; no random assignment to shock strategy	Intervention classification clear, protocol well described	EMS unblinded, field conditions lead to variations	Outcome data complete	Objective outcomes (VF termination and ROSC) measured consistently	Serious
Mapp et al. (2019)	Major: DSED only used in refractory cases	Matching present, reducing but not eliminating selection bias; EMS crews chose DSED through clinical judgment	Intervention classification clear, protocol well described	EMS unblinded, field conditions lead to variations	Outcome data largely complete; minimal missing data on ROSC and VF termination	Objective outcomes (survival to admission, ROSC, survival to discharge, neuro outcomes) measured consistently	Serious
Emmerson et al. (2017)	Major: DSED only used in refractory cases	Retrospective EMS selection; no random assignment to shock strategy	Intervention classification clear, protocol well described	EMS unblinded, field conditions lead to variations	Outcome data complete	Objective outcomes (ROSC, survival to hospital admission, survival to hospital discharge) measured consistently	Serious
Ross et al. (2016)	Major: DSED only used in refractory cases	Retrospective EMS selection; no random assignment to shock strategy	Intervention classification clear, protocol well described	EMS unblinded, field conditions lead to variations	Outcome data complete	Objective outcomes (ROSC, survival to hospital admission, survival to hospital discharge, neuro outcomes) measured consistently	Serious
Bjerregaard et al. (1999)	Major: Dual External DCCV used selectively for prior failed cardioversion	Retrospective selection, decisions based on refractoriness of AF	Intervention classification clear, protocol well described	Minimal deviations from protocol, performed in well‐controlled EP lab	Outcome data complete	Objective outcome (cardioversion success) measured consistently	Serious

**TABLE B3:** AMSTAR‐2 quality appraisal of included meta‐analyses. All included studies used ROBINS‐I for risk‐of‐bias assessment and none of the included studies assessed publication bias formally, so those two columns have been omitted.

Study	Protocol registered	Search comprehensiveness	Meta‐analytic methods	RoB considered in interpretation	Overall AMSTAR‑2 confidence
Yu et al. (2024)	No protocol registered	Multiple databases including PubMed, Embase, and Cochrane Library	Used random‐effects models, appropriate pooling, and correct handling of heterogeneity	Partial through incorporation of ROBINS‐I, but not integrated	Low Confidence
Abuelazm et al. (2023)	No protocol registered	Multiple databases including PubMed, Web of Science, Scopus, Embase, and Cochrane Library	Used network meta‐analysis and random‐effects modeling. Stated assumptions for transitivity and consistency and handled multi‐arm comparisons correctly.	Partial through incorporation of ROBINS‐I, but not integrated	Low Confidence
Miraglia et al. (2020)	No protocol registered	Multiple databases including PubMed, Embase, Scopus, and Cochrane Library	Used random‐effects models, appropriate pooling, correct handling of heterogeneity, and standard meta‐analytic stats	Partial through incorporation of ROBINS‐I, but not integrated	Low Confidence
Delorenzo et al. (2019)	No protocol registered	Multiple databases including Ovid Medline, Embase, CINAHL, Scopus, and Cochrane Library	Used random‐effects models, appropriate pooling, correct handling of heterogeneity, and standard meta‐analytic stats	Partial through incorporation of ROBINS‐I, but not integrated	Low Confidence
