# Supplementary material for: Double Consecutive Electrical Countershock for Atrial and Ventricular Fibrillation: A Narrative Critical Review
Source: Ann Noninvasive Electrocardiol. 2026 Nov 2;31(6):e70251. doi: 10.1111/anec.70251 (PMC13725440; doi:10.1111/anec.70251)
Supplement: Supplementary file 1 [file ANEC-31-e70251-s001.docx]

**Supplementary Table S1:** Studies excluded at the full-text stage with exclusion justifications

| **Full Study Reference (AMA)** | **Exclusion Category** | **Specific Reason for Exclusion** |
| --- | --- | --- |
| CTRI/2026/01/102381. A Study Comparing Two Methods of Defibrillation Used During Cardiac Arrest. https://trialsearchwhoint/Trial2aspx?TrialID=CTRI/2026/01/102381Published online 2026. doi:10.1002/central/CN-02965621 | Study in progress | Currently in progress, results and manuscript unavailable |
| NCT03249948. DOuble SEquential External Defibrillation for Refractory VF Pilot Study. https://clinicaltrialsgov/show/NCT03249948Published online 2017. doi:10.1002/central/CN-01495745 | Wrong publication type | Pilot study protocol |
| NCT07174986. Early Double Sequential Defibrillation in Out of Hospital Cardiac Arrest. https://clinicaltrialsgov/study/NCT07174986Published online 2025. doi:10.1002/central/CN-02914722 | Study in progress | Currently in progress, results and manuscript unavailable |
| Smida T, Crowe R, Drennan IR, et al. Use of Double Sequential External Defibrillation in the United States Before and After the DOSE VF Randomized Clinical Trial. Prehospital Emergency CarePublished online May 29, 2026:1-8. doi:10.1080/10903127.2026.2674263 | Wrong study design | Looked at use of DSED but not its effectiveness |
| Cheskes S, Drennan IR, Turner L, et al. The impact of defibrillation current and pad position on return of spontaneous circulation during refractory ventricular fibrillation. ResuscitationPublished online March 17, 2026:111061. doi:10.1016/j.resuscitation.2026.111061 | Wrong study design | Focused on pad positioning in DSED rather than effectiveness |
| Ulmanova L, Morena A, Citterio B, et al. Challenges in the treatment of out-of-hospital cardiac arrest. Trends in Cardiovascular Medicine2026;36(7):449-459. doi:10.1016/j.tcm.2026.02.011 | Wrong publication type | Editorial |
| Verkaik BJ, Walker RG, Taylor TG, et al. Refractory Ventricular Fibrillation in Out-of-Hospital Cardiac Arrest: Shock Characteristics, Predictors and Clinical Outcomes. Published online 2026:6478728. doi:10.2139/ssrn.6478728 | Wrong study design | Looked at predictors of refractory VF |
| Restoring Sinus Rhythm Using Dual-Shock Technique in Patients With Atrial Fibrillation. Clinicaltrials.ggJuly 28, 2026. Accessed October 9, 2026. https://clinicaltrials.gg/study/NCT07560644 | Study in progress | Currently in progress, results and manuscript unavailable |
| Nichol G, Atkins DL, Koster RW, et al. Scientific Priorities Related to the Use of Double Sequential External Defibrillation in Patients With Refractory Cardiac Arrest: Report From a Multistakeholder Thinktank. Journal of the American Heart Association2025;14(21):e044130-e044130. doi:10.1161/jaha.125.044130 | Wrong study design | Thinktank report |
| Alexandrou K, Khattab E, Asimakopoulou E. Enhancing Patient Safety in Refractory Ventricular Fibrillation: A Systematic Review of Double Sequential and Vector Change Defibrillation Barriers. Healthcare2025;13(20):2645. doi:10.3390/healthcare13202645 | Wrong publication type | Systemic review without meta-analysis |
| Bharwani S, Chung MK, Morin DP. Strategies for optimizing efficacy of electrical cardioversion of atrial fibrillation. Progress in Cardiovascular DiseasesPublished online June 30, 2025. doi:10.1016/j.pcad.2025.06.009 | Wrong publication type | Systemic review without meta-analysis |
| Cheskes S, Dorian P, Drennan IR, Scales DC. Double Sequential External Defibrillation for Refractory Ventricular Fibrillation: A Perspective on the Canadian Cardiovascular Society Practice Update. Canadian Journal of Cardiology2025;41(4):792. doi:10.1016/j.cjca.2024.05.011 | Wrong study design | Editorial |
| Ao CV, Ho MP. Double defibrillation for patients with refractory ventricular fibrillation. The American Journal of Emergency Medicine2025;88:233. doi:10.1016/j.ajem.2024.09.054 | Wrong publication type | Report |
| Blick Nordkvist E, Awad A, Riva G, et al. Early double sequential defibrillation - a randomized pilot study. European Heart Journal2025;46(Supplement_1). doi:10.1093/eurheartj/ehaf784.2189 | Wrong publication type | Pilot study focused on EMS provider practices rather than effectiveness |
| Derkenne C, Cazes N, Javaudin F, et al. Refractory ventricular fibrillation: what defibrillation strategy? Annales françaises de médecine d’urgence2025;15(6):397-407. doi:10.1684/afmu.2025.0680 | Foreign language | Only officially available in French |
| Ramzy M, Hughes PG. Double Defibrillation. PubMed2024. Accessed October 9, 2026. https://pubmed.ncbi.nlm.nih.gov/31334951/ | Wrong publication type | Textbook section |
| White B. Double Sequential Defibrillation: An Update of Contemporary Defibrillation Strategies for Pulseless Ventricular Tachycardia and Fibrillation. CureusPublished online January 12, 2025. doi:10.7759/cureus.77316 | Wrong publication type | Editorial |
| Verkaik BJ, Walker RG, Taylor TG, et al. Defibrillation and refractory ventricular fibrillation. European Heart Journal2024;46(6):582-584. doi:10.1093/eurheartj/ehae767 | Wrong therapy | Looked only at standard defibrillation |
| Stub D, Batchelor R, Ball J. Double or nothing: Alternative defibrillation strategies reduces downtime in ventricular fibrillation. Resuscitation2025;209:110570. doi:10.1016/j.resuscitation.2025.110570 | Wrong publication type | Editorial |
| Nordviste V, Marius R, Krüger AJ, Rødseth BJ. Time difference between pad placement in single versus double external defibrillation: A live patient simulation model. Resuscitation Plus2024;19:100741. doi:10.1016/j.resplu.2024.100741 | Wrong publication type | Examined EMS readiness to perform DSED in a training environment |
| Cheskes S, Dorian P, Drennan IR, Scales DC. Double Sequential External Defibrillation for Refractory Ventricular Fibrillation: A Perspective on the Canadian Cardiovascular Society Practice Update. Canadian Journal of Cardiology2025;41(4):792. doi:10.1016/j.cjca.2024.05.011 | Wrong publication type | Letter to the editor |
| Cuomo A, de Menezes LM, Elli S, Rocchi B, Rocco M, Viola S. Is it time to update our defibrillation strategies for refractory ventricular fibrillation? Internal and Emergency Medicine2024;19(3):835-837. doi:10.1007/s11739-023-03504-4 | Wrong publication type | Research update/summary |
| Rahimi M, Drennan IR, Turner L, Dorian P, Cheskes S. The impact of double sequential shock timing on outcomes during refractory out-of-hospital cardiac arrest. Resuscitation2024;194:110082. doi:10.1016/j.resuscitation.2023.110082 | Wrong outcome | Evaluated DSED intervals |
| Ali R, Barsoum B, Lago R. Abstract Mo035: Double (Dual) Sequential Defibrillation Versus Standard Defibrillation for Refractory Ventricular Fibrillation: A Systematic Review and Meta-Analysis. Circulation Research2024;135(Suppl_1):AMo035-AMo035. doi:10.1161/res.135.suppl_1.mo035 | Wrong publication type | Abstract |
| A Pilot Study Assessing the Feasibility of a Randomized Trial Comparing the Effect of Early Double Sequential Defibrillation as Soon as Possible in OHCA With a Shockable Rhythm (VT/VF) Compared to Standard Defibrillation. EmbaseJanuary 1, 2021. https://www.embase.com/search/results?subaction=viewrecord&id=LNCT06447805&from=export | Study in progress | Study was in progress when this was published, completed study included in manuscript |
| Ali R, Barsoum B, Lago R. Abstract Mo035: Double (Dual) Sequential Defibrillation Versus Standard Defibrillation for Refractory Ventricular Fibrillation: A Systematic Review and Meta-Analysis. Circulation Research2024;135(Suppl_1):AMo035-AMo035. doi:10.1161/res.135.suppl_1.mo035 | Wrong publication type | Abstract, duplicate missed in earlier stages of screening |
| Perna B, Guarino M, De Fazio R, et al. Alternative Defibrillation Strategies for Refractory Ventricular Fibrillation: A Systematic Review and Meta-Analysis. Published online September 29, 2023. doi:10.1101/2023.09.26.23296188 | Study in progress | Preprint, not peer-reviewed |
| Aymond JD, Gullatt T, Bernard ML, et al. CE-452778-3 EFFICACY AND SAFETY OF DUAL DIRECT CURRENT CARDIOVERSION VERSUS SINGLE DIRECT CURRENT CARDIOVERSION AS AN INITIAL TREATMENT STRATEGY IN OBESE PATIENTS WITH ATRIAL FIBRILLATION. Heart Rhythm2023;20(5):S69-S70. doi:10.1016/j.hrthm.2023.03.347 | Wrong publication type | Abstract |
| Ao CV, Ho MP. Double sequential defibrillation for refractory ventricular fibrillation. The American Journal of Emergency Medicine2023;70:187. doi:10.1016/j.ajem.2023.06.047 | Wrong publication type | Textbook section, duplicate missed in earlier stages of screening |
| Abuelazm M, Ghanem A, Katamesh BE, et al. PO-05-008 NETWORK META-ANALYSIS OF THE COMPARATIVE EFFICACY OF DOUBLE SEQUENTIAL EXTERNAL, VECTOR-CHANGE, AND STANDARD DEFIBRILLATION FOR REFRACTORY VENTRICULAR FIBRILLATION OUT-OF-HOSPITAL CARDIAC ARREST. Heart Rhythm2023;20(5):S697. doi:10.1016/j.hrthm.2023.03.1449 | Wrong publication type | Abstract |
| Taverne LF, Koster RW, de Jong JSSG. Defibrillation techniques in persistent ventricular fibrillation: which is the most effective? Ned Tijdschr Geneeskd2023;167:D7592. Accessed October 9, 2026. https://pubmed.ncbi.nlm.nih.gov/37742120/ | Foreign language | Only officially available in Dutch |
| Cheskes S, McLeod S, Scales DC. Double sequential external defibrillation for refractory ventricular fibrillation. Intensive Care Medicine2023;49(4). doi:10.1007/s00134-023-06993-1 | Wrong publication type | Research update/summary |
| Lim GB. Alternative defibrillation strategies improve outcomes. Nature Reviews Cardiology2022;20(1):5-5. doi:10.1038/s41569-022-00808-4 | Wrong publication type | Research update/summary |
| Defibrillation Strategies for Refractory Ventricular Fibrillation. New England Journal of Medicine2023;388(9):860-863. doi:10.1056/nejmc2216382 | Wrong publication type | Correspondance |
| Defibrillation Strategies for Refractory Ventricular Fibrillation. New England Journal of Medicine2023;388(9):860-863. doi:10.1056/nejmc2216382 | Wrong publication type/Duplicate | Duplicate missed in earlier stages of screening |
| Izzati MAF, Sulon ARZR, Hamad AH. Electrical storm – The double down. Visual Journal of Emergency Medicine2022;26:101228. doi:10.1016/j.visj.2021.101228 | Wrong therapy | Discussion of general treatment for refractory VF |
| Li Y, He X, Li Z, Li D, Yuan X, Yang J. Double sequential external defibrillation versus standard defibrillation in refractory ventricular fibrillation: A systematic review and meta-analysis. Frontiers in Cardiovascular Medicine2022;9. doi:10.3389/fcvm.2022.1017935 | Wrong study design | Systemic review and meta-analysis but without primary source data |
| Cheskes S, Hunter M, Drennan I. Just the facts: double sequential external defibrillation for refractory ventricular fibrillation. Canadian Journal of Emergency Medicine2021;23(2):156-158. doi:10.1007/s43678-020-00039-x | Wrong publication type | Research update/summary |
| Roach C, Tainter CR, Sell RE, Wardi G. Resuscitating Resuscitation: Advanced Therapies for Resistant Ventricular Dysrhythmias. The Journal of Emergency Medicine2021;60(3):331-341. doi:10.1016/j.jemermed.2020.10.051 | Wrong study design | Research update/summary |
| Dyer S, Mogni B, Gottlieb M. Electrical storm: A focused review for the emergency physician. The American Journal of Emergency Medicine2020;38(7):1481-1487. doi:10.1016/j.ajem.2020.04.017 | Wrong therapy | Looked only at standard defibrillation |
| Miraglia D, Miguel LA, Alonso W, Ayala JE. Double sequential defibrillation for out-of-hospital refractory ventricular fibrillation: A scoping review. The American Journal of Emergency Medicine2020;38(6):1211-1217. doi:10.1016/j.ajem.2019.12.047 | Wrong study design | Scoping review |
| Prehospital Double Defibrillation for Refractory Ventricular Fibrillation: A Scoping Review Protocol. Innovationsincrm.com2026. Accessed October 9, 2026. https://www.innovationsincrm.com/cardiac-rhythm-management/articles-2020/june/1606-prehospital-double-defibrillation | Wrong study design | Scoping review protocol |
| Hamilton RJ, Ramzy M, Teufel J, Laub G, Kresh JY. Dual Defibrillation is Highly Variable: An Analysis of Pulse Interval Delivered in Dual Defibrillation. Prehospital Emergency Care2019;24(2):232-237. doi:10.1080/10903127.2019.1621411 | Wrong study design | Examined timing metrics on the practice of DSED in the field |
| Hamilton RJ, Ramzy M, Teufel J, Laub G, Kresh JY. Dual Defibrillation is Highly Variable: An Analysis of Pulse Interval Delivered in Dual Defibrillation. Prehospital Emergency Care2019;24(2):232-237. doi:10.1080/10903127.2019.1621411 | Wrong study design/Duplicate | Duplicate missed in earlier stages of screening |
| Bero M, Sochor M, Wong S, Brady W. Changing the management of refractory ventricular fibrillation: The consideration of earlier utilization of dual sequential defibrillation. The American Journal of Emergency Medicine2020;38(3):545-548. doi:10.1016/j.ajem.2019.05.048 | Wrong publication type | Summary of case reports |
| Kumar V, Yamane D, Aljohani B, Alsabban A, Pourmand A. Double sequential external defibrillation. Resuscitation2020;152:212-213. doi:10.1016/j.resuscitation.2020.05.015 | Wrong publication type | Letter to the editor |
| Drennan IR, Dorian P, McLeod S, et al. DOuble SEquential External Defibrillation for Refractory Ventricular Fibrillation (DOSE VF): study protocol for a randomized controlled trial. Trials2020;21(1). doi:10.1186/s13063-020-04904-z | Wrong publication type | Study protocol, full publication included in manuscript |
| Berg KM, Soar J, Andersen LW, et al. Adult Advanced Life Support: 2020 International Consensus on Cardiopulmonary Resuscitation and Emergency Cardiovascular Care Science With Treatment Recommendations. Circulation2020;142(16_suppl_1). doi:10.1161/cir.0000000000000893 | Wrong publication type | Review without meta-analysis |
| Deakin CD. Dual sequential defibrillation: Hold your horses! Resuscitation2020;150:189-190. doi:10.1016/j.resuscitation.2020.03.001 | Wrong publication type | Editorial |
| Clemency BM, Pastwik B, Dennison G. Double sequential defibrillation and the tyranny of the case study. The American Journal of Emergency Medicine2019;37(4):792-793. doi:10.1016/j.ajem.2018.09.002 | Wrong publication type | Editorial |
| Taylor TG, Melnick SB, Chapman FW, Walcott GP. An investigation of inter-shock timing and electrode placement for double-sequential defibrillation. Resuscitation2019;140:194-200. doi:10.1016/j.resuscitation.2019.04.042 | Wrong population | Examined incidence of defibrillator damage in DSED |
| Double Sequence External Defibrillation: A Randomized Controlled Trial in Patients With Atrial Fibrillation Refractory to DC Cardioversion. EmbaseJanuary 1, 2019. https://www.embase.com/search/results?subaction=viewrecord&id=LNCT03827915&from=export | Wrong publication type | Study protocol |
| Dual Shock Versus Single Shock Synchronized External Direct Current Cardioversion for Atrial Fibrillation - A Double-blinded Randomized Trial. January 1, 2018. https://www.embase.com/search/results?subaction=viewrecord&id=LNCT03943693&from=export | Wrong publication type | Study protocol |
| Cheskes S, Wudwud A, Turner L, et al. LO07: Double sequential external defibrillation improves termination of ventricular fibrillation and return of spontaneous circulation in shock-refractory out-of- hospital cardiac arrest. CJEM2019;21(S1):S9. doi:10.1017/cem.2019.50 | Wrong publication type | Review without meta-analysis |
| Pourmand A, Galvis J, Yamane D. The controversial role of dual sequential defibrillation in shockable cardiac arrest. The American Journal of Emergency Medicine2018;36(9):1674-1679. doi:10.1016/j.ajem.2018.05.078 | Wrong publication type | Review without meta-analysis |
| Simon EM, Tanaka K. Double Sequential Defibrillation. Cardiology Clinics2018;36(3):387-393. doi:10.1016/j.ccl.2018.03.006 | Wrong publication type | Review without meta-analysis |
| Davis M, Schappert A, Van Aarsen K, Loosley J, McLeod S, Cheskes S. P029: A descriptive analysis of defibrillation vector change for prehospital refractory ventricular fibrillation. CJEM2018;20(S1):S67-S67. doi:10.1017/cem.2018.227 | Wrong therapy | Examined treatment for refractory VF in general |
| Late-Breaking Science Abstracts From the American Heart Association’s Scientific Sessions 2018 and Late-Breaking Abstracts in Resuscitation Science From the Resuscitation Science Symposium 2018. Circulation2018;138(25). doi:10.1161/cir.0000000000000636 | Wrong publication type | Abstract |
| Emergency Medicine. Double Sequential Defibrillation for Refractory Ventricular Fibrillation and Pulseless Ventricular Tachycardia \| MDedge. Mdedge.com2017. Accessed October 9, 2026. https://mdedge.com/emed-journal/article/151602/cardiology/double-sequential-defibrillation-refractory-ventricular | Wrong study design | Case series |
| Emergency Medicine. Double Sequential Defibrillation for Refractory Ventricular Fibrillation and Pulseless Ventricular Tachycardia \| MDedge. Mdedge.com2017. Accessed October 9, 2026. https://mdedge.com/emed-journal/article/151602/cardiology/double-sequential-defibrillation-refractory-ventricular | Wrong study design/Duplicate | Case series |
| Cortez E, Krebs W, Davis J, Keseg DP, Panchal AR. Use of double sequential external defibrillation for refractory ventricular fibrillation during out-of-hospital cardiac arrest. Resuscitation2016;108:82-86. doi:10.1016/j.resuscitation.2016.08.002 | Wrong study design | Retrospective chart review, assessed the wrong outcomes |
| Deakin CD, Kerber RE. Dual sequential defibrillation: Does one plus one equal two? Resuscitation2016;108:A1-A2. doi:10.1016/j.resuscitation.2016.08.013 | Wrong publication type | Editorial |
| Stuart Gerstein N, Shah MB, Jorgensen KM. Erratum to “Simultaneous Use of Two Defibrillators for the Conversion of Refractory Ventricular Fibrillation” J Cardiothorac Vasc Anesth 29(2015):369-378. Journal of Cardiothoracic and Vascular Anesthesia2015;29(4):e39. doi:10.1053/j.jvca.2015.05.196 | Wrong publication type | Erratum |
| Gerstein NS, Shah MB, Jorgensen KM. Simultaneous Use of Two Defibrillators for the Conversion of Refractory Ventricular Fibrillation. Journal of Cardiothoracic and Vascular Anesthesia2015;29(2):421-424. doi:10.1053/j.jvca.2013.10.016 | Wrong study design | Case report |
| Botto GL, Boriani G, Favale S, et al. Treatment of atrial fibrillation with a dual defibrillator in heart failure patients (TRADE HF): protocol for a randomized clinical trial. Trials2011;12(1). doi:10.1186/1745-6215-12-44 | Wrong therapy | Examines cardiac resynchronization devices |
| Velázquez Rodríguez E, Martínez Enríquez A, Cancino Rodríguez C, Olvera Morales G, Rangel Rojo J, Arias Estrada S. Double sequential electrical transthoracic shocks for refractory atrial fibrillation. Arch Cardiol Mex2005;75 Suppl 3:S3-69-80. Accessed October 9, 2026. https://pubmed.ncbi.nlm.nih.gov/16366172/ | Foreign language | Only officially available in Spanish |
| Ricci R, Quesada A, Pignalberi C, et al. Dual Defibrillator Improves Quality of Life and Decreases Hospitalizations in Patients with Drug Refractory Atrial Fibrillation. Journal of Interventional Cardiac Electrophysiology2004;10(1):85-92. doi:10.1023/b:jice.0000011490.32755.40 | Wrong therapy | Examines dual defibrillator devices |
| Misier AR, Beukema WP, Willems R. Multisite Atrial Pacing for Atrial Fibrillation Prevention: Where to Go from Here? Cardiac Electrophysiology Review2003;7(4):329-332. doi:10.1023/b:cepr.0000023132.72125.ca | Wrong therapy | Examines multi-site atrial pacing devices |
| MARROUCHE NF, BARDY GH, FRIELITZ HJ, GÜNTHER J, BRACHMANN J. Quadruple Pads Approach for External Cardioversion of Atrial Fibrillation. Pacing and Clinical Electrophysiology2001;24(9):1321-1324. doi:10.1046/j.1460-9592.2001.01321.x | Wrong therapy | Consecutive, spread out shocks rather than proper dual-DCCV |
| Prakash A, Saksena S, Krol RB, Philip G. Right and left atrial activation during external direct-current cardioversion shocks delivered for termination of atrial fibrillation in humans. The American Journal of Cardiology2001;87(9):1080-1088. doi:10.1016/s0002-9149(01)01465-5 | Wrong therapy | Wrong type of cardioversion protocol, not dual-DCCV |
| Hoch DH, Batsford WP, Greenberg SM, et al. Double sequential external shocks for refractory ventricular fibrillation. Journal of the American College of Cardiology1994;23(5):1141-1145. doi:10.1016/0735-1097(94)90602-5 | Wrong population | Patients with induced VF in the inpatient elecrtophysiology lab |
| Bardy GH, Ivey TD, Allen MD, Johnson G, Greene HL. Prospective comparison of sequential pulse and single pulse defibrillation with use of two different clinically available systems. Journal of the American College of Cardiology1989;14(1):165-171. doi:10.1016/0735-1097(89)90068-5 | Wrong therapy | Examines intraoperative defibrillation |
